# Farmers’ Cohort for Agricultural Work-Related Musculoskeletal Disorders (FARM) Study: Study Design, Methods, and Baseline Characteristics of Enrolled Subjects

**DOI:** 10.2188/jea.JE20140271

**Published:** 2016-01-05

**Authors:** Hannae Jo, Sora Baek, Hee-won Park, Sang-Ah Lee, Jiyoung Moon, Jae E. Yang, Ki Sung Kim, Jee Yong Kim, Eun Kyoung Kang

**Affiliations:** 1 Center for Farmers’ Safety and Health, Kangwon National University Hospital, Chuncheon-si, Gangwon-do, South Korea; 2 Department of Rehabilitation Medicine, Kangwon National University Hospital, Chuncheon-si, Gangwon-do, South Korea; 3 Department of Rehabilitation Medicine, School of Medicine, Kangwon National University, Chuncheon-si, Gangwon-do, South Korea; 4 Gangwon Do Rehabilitation Hospital, Chuncheon-si, Gangwon-do, South Korea; 5 Department of Preventive Medicine, School of Medicine, Kangwon National University, Chuncheon-si, Gangwon-do, South Korea; 6 Department of Preventive Medicine, Kangwon National University Hospital, Chuncheon-si, Gangwon-do, South Korea; 7 Department of Biological Environment, Kangwon National University, Chuncheon-si, Gangwon-do, South Korea; 8 Department of Regional Infrastructure Engineering, Kangwon National University, Chuncheon-si, Gangwon-do, South Korea; 9 NongTeo Co., Ltd., Chuncheon-si, Gangwon-do, South Korea

**Keywords:** farmer, agriculture, work, musculoskeletal disorders, health

## Abstract

**Background:**

The ongoing Farmers’ Cohort for Agricultural Work-related Musculoskeletal Disorders (FARM) study was developed to evaluate health status and related factors in farmers.

**Methods:**

Farmers in Kangwon Province, South Korea, were recruited. Baseline characteristics were determined using questionnaires about sociodemographic and health characteristics and agricultural work-related factors. In addition, laboratory examinations (lumbar spinal radiography and serologic testing) were conducted.

**Results:**

The FARM study covers eight rural areas and recruited 1013 subjects (534 women; mean [standard deviation {SD}] age, 57.2 [7.5] years). Musculoskeletal pain in multiple areas was reported by 925 subjects (91.3%), and low back pain (63.8%) was the most frequent site of pain. Farmer’s Stress Inventory (mean [SD], 77.7 [10.2]; range, 28–112] and subjective stress index (mean [SD], 5.3 [2.4]; range, 0–10) were above median scale values, reflecting a stressful condition, while the EuroQol-5D-3L index and the EuroQol-Visual Analog Scale scores were high (mean [SD], 0.9 [0.1]; range −0.171–1 and mean [SD], 67.7 [18.7]; range 0–100, respectively), reflecting good life quality. In total, 53% of participants had worked in farming for more than 30 years, and workers involved in dry-field farming comprised the largest subgroup (41.5%). Most participants (94.3%) had no more than a high school education, and families with annual income below 20 million won constituted the largest subgroup (36.3%).

**Conclusions:**

The FARM study may provide data on the current health status and related sociodemographic and agricultural work-related risk factors in Korean farmers, with the goal of providing a scientific basis for developing coping interventions and preventive strategies.

## INTRODUCTION

Musculoskeletal disorders (MSDs) are common among farmers because of the extremely labor-intensive workload in agriculture.^1^^–^^3^ The estimated lifetime prevalence of MSDs among farmers has been reported as 90.6%, and the 1-year MSD prevalence has been reported as 76.9%.^4^ In population-based studies, MSDs were more frequent among farmers, with more severe symptoms affecting the hands and forearms, low back, and hips compared to less physically demanding non-farmer occupations.^5^^–^^7^ The impact of MSDs in farmers is substantial and results in long-term disability and income loss. Chapman and Meyers reported that MSDs occur over 20 times more frequently than pesticide injuries and illness in United States agriculture, and MSDs have cost the American farming industry in excess of $167 million for reported injuries.^8^^,^^9^ Additionally, Kirkhorn et al. reported that agricultural workers are at particular risk of arthritis-related disability.^10^

However, while MSDs in farmers are the cause of significant health problems and result in loss of productivity, previous studies have only demonstrated the higher frequency of MSDs and the impact of workload on MSDs in farmers and have only followed participants for limited durations. Validated longitudinal information about overall agricultural work-related health status and risk factors related to MSDs is urgently needed to improve the health status of farmers. We hypothesized that there might be serial changes in MSDs that are affected by agricultural and work-related factors.

The purpose of our study was to elucidate the sociodemographic conditions, health characteristics (agricultural work-related stress, frailty, musculoskeletal pain, quality of life, and back pain-related disability), and associated work-related factors, such as agricultural conditions and work-related ergonomic risk, in Korean farmers through long-term follow up.

## METHODS

### Subjects

The Farmers’ Cohort for Agricultural Work-Related Musculoskeletal Disorders (FARM) study was conducted by the Center for Farmers’ Safety and Health at Kangwon National University Hospital. We first selected 1822 farmers (aged 40–70 years) who owned or rented a farm in Kangwon Province in South Korea (including Chuncheon, Hongcheon, Hwacheon, Yang-gu, Injae, Cheorwon, Jeongseon, and Seogok) through the agricultural cooperative units. Kangwon Providence had a population of 1.46 million people in 2010, of which 13.1% are farmers (compared to the national average of 6.4%).^11^

Of the 1822 farmers initially selected, 795 did not participated in our study (586 refusals, 190 unobtainable phone numbers, 9 traveling to other areas, 8 aged ≥70 years, 1 hospitalization, and 1 immobilization). We recruited the remaining 1027 participants from September 2013 through June 2014. Our research team visited each agricultural cooperative unit and conducted the baseline study. The participants were then verified as official farmers by the local representatives in the National Agricultural Cooperative Federation. Finally, amputees (*n* = 4), nonagricultural workers (*n* = 9), and very-low-weight (*n* = 1) subjects were excluded, leaving 1013 included participants in this prospective cohort study (Figure).

**Figure.**
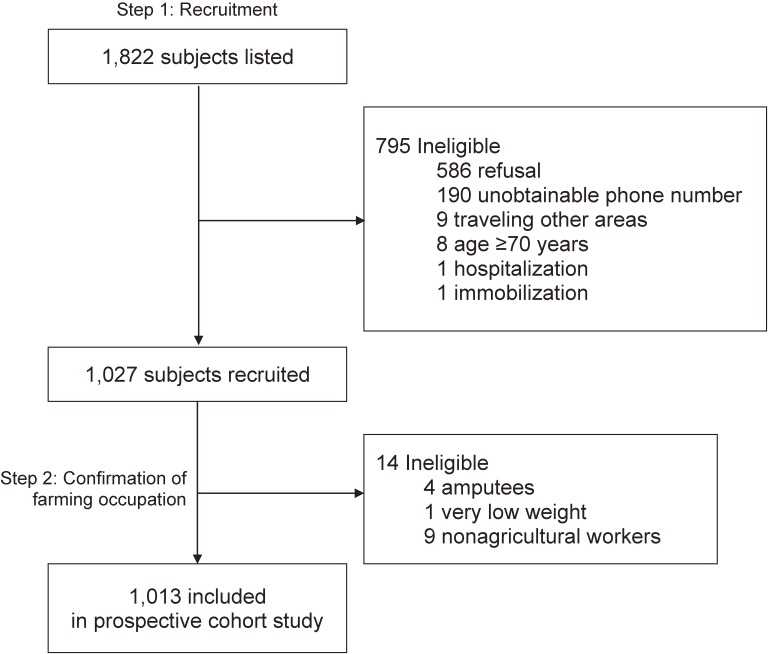
Inclusion flowchart.

### Standard protocol approvals, registrations, and patient consent

This study was approved by the Institutional Review Board of the Kangwon National University Hospital (IRB No. 2013-06-009-007) and Clinical Research Information Service of Korean National Institutes of Health (ID: KCT0000829) which became a member of the World Health Organization’s International Clinical Trials Registry Platform. Additionally, written informed consent was obtained from all participants in the study.

### Questionnaires

#### Sociodemographic conditions

Questionnaires of sociodemographic conditions asked about age, sex, composition of family living together with respondent (number of members and their family relations), marital status, level of education, annual income, occupational history, systemic medical history, family medical history, history of smoking and drinking, and dietary habits (frequency of consumption of each of the foods listed). For women, reproductive history was also obtained.

#### Health characteristics

Table 1 shows the baseline health characteristics of participants. Agricultural stress was measured using the short form of the Farm Stressor Inventory, which consists of eight domains and 28 questions scored on a 4-point scale.^12^ In addition, stress response was also evaluated using a subjective scoring method.

**Table 1.** Evaluated health characteristics

Questionnaire
Farmer’s stress inventory (28–112: scored on a 4-point scale, 1 = strongly disagree to 4 = strongly agree)
Labor intensity (amount of work, manpower, time management, chores, work in time)
Job environment (outside work, uncomfortable posture, crop-dusting, repetition of same task)
Job esteem and social reputation (self-esteem, worth)
Physical environment and weather condition (weather, insects/diseases, natural disasters)
Financial problems (income, debt, economic issues, revenue losses, regularity of income, price)
Uncertainty (uncertain future, career transition)
Government policy (free trade, loans, cost for farming materials, distribution structure)
Health problems (health worries, sickness)
Subjective stress index (0–10: 0 = no stress to 10 = maximum stress)
Korean Frailty Index (0–8: scored with 0 or 1, score 1 indicates less frailty)
1. How many times did you require admission to hospital during the last year?
2. How healthy would you say you are?
3. Do you take more than 4 kinds of drugs regularly?
4. Have you lost a lot of weight during the last year (enough that clothes become loose)?
5. Have you felt depressed or sad during the last month?
6. Have you experienced incontinence of urine or stool during the last month?
7. Timed up and go test
8. Have you experienced problems in your daily life due to poor hearing or vision?
Self-reported questionnaire developed by the Korean Occupational Safety and Health Agency
General condition
Leisure activity (at least 30 minutes per session and more than 2 days a week)
Household work
Medical disease history
History of prior injury
Level of physical burden
Musculoskeletal pain
Location (neck, shoulder, arm/elbow, hand/wrist/finger, low back, leg/foot/toe)
Laterality (right, left, or bilateral)
Duration (<1 day, <1 week, <1 month, <6 month, and ≥6 month)
Severity (mild, moderate, severe, or very severe)
Frequency (semiannually, quarterly, monthly, weekly, or daily)
Presence of pain within 1 week
Consequence of pain (visit clinic, visit pharmacy, sick leave, change job, do nothing, or other)
EuroQol-5D-3L (three levels of functioning: no problems, some problems, and unable/extreme problems)
Do you have problems in walking about?
Do you have problems with washing or dressing yourself?
Do you have problems with performing your usual activities (work, study, housework, family or leisure activities)?
Do you have pain or discomfort?
Are you anxious or depressed?
EuroQol-Visual Analog Scale (0–100: 0 = the worst imaginable health state, 100 = the best imaginable health state)

To evaluate disability associated with back pain, the self-administered Oswestry Disability Index was used.^13^ The Korean Frailty Index, which was created by a panel of experts from the Korean Geriatrics Society, was used to estimate frailty^14^; the scale includes eight items scored with 0 or 1, and scores of 5–8 were defined as frail, scores of 3–4 as pre-frail, and scores of 0–2 as normal.

To evaluate the musculoskeletal condition, we used a structured self-reported questionnaire, which was developed by the Korean Occupational Safety and Health Agency.^15^ The questionnaire assessed the general musculoskeletal condition and musculoskeletal pain characteristics, as well as consequences of pain during last year.

We used the EuroQol-5D-3L (EQ-5D-3L) to measure health-related quality of life and stratified respondents into three levels of functioning (no problems, some problems, or extreme problems). The EQ-5D-3L index was calculated using a previously suggested formula.^16^ Additionally, respondents rated their current health status according to the EuroQol-Visual Analog Scale (EQ-VAS) from 0 (worst imaginable health) to 100 (best imaginable health) (a detailed list of the evaluated health status items are listed in Table 1).

#### Agricultural work-related factors

Table 2 lists the surveyed items regarding agricultural work-related factors. The respondents completed four separate questionnaires regarding agricultural work. For general agricultural conditions, questions were asked about crops, cultivated land, working conditions, and machinery. The agricultural work-related accident questionnaire asked about the location of work-related injuries (the same body parts as the MSDs questionnaire) and the types of farming work related to the accident. The agricultural care level, which assesses farming and living environments, was evaluated using an assessment tool developed by the Korean Rural Development Administration.^17^ Self-reported agricultural work-related ergonomic risk was investigated using an ergonomic checklist developed by the Korean Rural Development Administration and will be verified by our study through official agreements for use of this checklist in this study. The checklist, composed of 20 items of agricultural work-related body positions, assesses the frequency (none, occasionally, frequently, or always) of adopting those positions per day.

**Table 2.** Evaluated questionnaires for agricultural work-related factors

Questionnaire
Agricultural condition
Three kinds of main crops
The crop producing the largest income
Total area of cultivated land
Extent and gradient of farmland
Daily working hours
Individual work proportion for total work
Number of breaks
Composition of physical activity
Proportion of machinery use
Three most frequently used machines associated with the 3 main crops
Agricultural work-related accident during
Machine use
Machine transfer
Machine check-up
Loading or transporting of agricultural products
Facilities check-up
Carrying agricultural products
Working in a high place
Agricultural care level (41–123: scores ranging from 1 to 3, higher score indicates a better level of environment)
1. Working environment (effort to improve working environment, width of working space passage, farming tool arrangement, working space organization, safety check, heating and cooling system, lighting, resting room)
2. Assistive equipment (assistive transfer device, worktable protective equipment for crop-dusting, general protective equipment like goggle, medicine chest)
3. Task management (task plan, division of works, time management, busy season distribution, labor mobilization for farming season, regular day-off)
4. Labor management (working hours per day during farming season, consecutive working hours, breathing time, dawn work (before 7 am), weight of package per transport, way of transport/working hours during extremely hot or cold weather, health condition check)
5. Work burden (fatigue degree after work, management after crop-dusting, number of pain existing region, proportion of labor intensive work, proportion of carefulness demanding work/proportion of bad posture during work, way to relax)
6. Lifestyle management (regular meal, nutrition, sleeping hours, leisure life, physical condition management during farming season, working uniform change, changing room)
Agricultural work-related ergonomic risk (four-point scale: none, occasionally, frequently, or always)
1. Shoulder flexion (45–90°)
2. Shoulder flexion (≥90°)
3. Pulling and pushing objects with stretched arm
4. Neck flexion (≥20°) or extension (≥5°)
5. Neck flexion (≥45°) or extension (≥20°)
6. Carrying heavy objects over one’s shoulder or head (≥25 kg more than 20 times a day or ≥50 kg more than 10 times a day)
7. Trunk flexion or twisting (20–45°)
8. Trunk flexion or twisting (≥45°)
9. Lifting up heavy objects (≥25 kg more than 10 times a day or ≥10 kg more than 25 times a day)
10. Pulling and pushing with excessive force
11. Exposure to whole body vibration while driving cultivator or tractor
12. Squatting
13. Going up and down ladder or jumping from height of more than two staircases
14. Striking knee more than 20 times a day
15. Highly repetitive wrist flexion (≥30°) or extension (≥45°)
16. Constant stress on finger or wrist (more than 0.9 kg for finger/more than 4.5 kg for wrist)
17. Exposure to local vibration from farm machines
18. Striking the back of hand more than 20 times a day
19. Repetitive elbow flexion and extension

### Laboratory examinations

#### Serologic tests

Blood samples (5 cc) were collected, kept in a portable refrigerator after centrifugation, and analyzed at Kangwon National University Hospital for biomarkers related to or predictive of agricultural work-related stress, frailty, and spinal degenerative changes, including C-reactive protein, vitamin D, and dehydroepiandrosterone sulfate.^18^^,^^19^

#### Radiographic tests

To determine quantitative biomechanical properties of the spine, anteroposterior, neutral lateral, lateral (flexion), and lateral (extension) lumbar radiographs were taken using a portable X-ray vehicle. Disc height change was evaluated using the relative percentage of the disc height of L4-5 and L5-S1 compared with adjacent discs and were graded as 0 (normal), 1 (mild, >75%), 2 (moderate, >50%), 3 (severe, >25%), or 4 (very severe, <25%). Additionally, an L5 osteophyte score was obtained by summing the points of osteophyte formation on eight edges of the vertebral body (no osteophytes = 0, <3 mm = 1 point, ≥3 mm = 2 points) and then graded as 0 (0 points), 1 (1–4 points), 2 (5–8 points), 3 (9–12 points), or 4 (13–16 points).^20^ Spondylolisthesis of L5 on S1, lumbar scoliosis, or spondylolysis, and a history of compression fracture or lumbar surgery, were also confirmed using radiographic findings.

### Follow-up surveys

A follow-up study will be conducted after 1 year, which will include in-depth medical evaluations focused on agricultural work-related musculoskeletal disease, such as spinal bone mineral density, lumbar body composition CT (to show the lumbar back muscle), back muscle strength test, and several performance tests (eg, grip strength, and walking speed). Of the subjects recruited for the primary survey, farmers with <30% of total activity as agriculture-related activity, <30% of total income as agricultural income, and <991.7 m^2^ cultivated land area will be excluded from the secondary survey because of their low representativeness for active farmers’ characteristics.

## RESULTS

Baseline characteristics are described in Table 3. Of 1013 subjects, 534 (52.7%) were women and 479 (47.3%) were men, and the mean (standard deviation [SD]) age was 57.2 (7.5) years. The majority of participants were married (91.1%), and 94.3% of the cohort had no more than a high school education. More than half (53.0%) had worked in farming for more than 30 years. The most common type of farming was dry field farming (41.5%), followed by greenhouse farming (30.7%), rice farming (15.5%), and orchard farming (12.3%). Regarding financial status, the largest subgroup comprised families with annual income below 20 million won (36.3%), followed by 20–30 million won (20.6%), 30–40 million won (14.1%), >50 million won (11.6%), and 40–50 million won (8.7%). Regarding smoking status, 35.7% of participants have smoked more than five packs of cigarettes during the preceding year, and 22.0% of participants were current smokers. The majority of participants had experience with alcohol drinking (63.5%), and 54.3% of participants reported current alcohol consumption (Table 3).

**Table 3.** Baseline characteristics of enrolled subjects (*n* = 1013)

Variable	
Mean (SD) age, years	57.2 (7.5)
<65 years	798 (78.8)
≥65 years	215 (21.2)
Men/Women	479/534 (47.3/52.7)
Mean (SD) height, cm	159.6 (8.8)
Mean (SD) weight, kg	64.6 (10.6)
Mean (SD) BMI, kg/m^2^	25.3 (3.2)
Low weight (BMI <18.5)	12.0 (1.2)
Normal weight (BMI 18.5–24.9)	458.0 (45.2)
Overweight (BMI 25.0–29.9)	468.0 (46.2)
Obesity (BMI >30)	75.0 (7.4)
Marital status	
Never married	16 (1.6)
Married	923 (91.1)
Separated	4 (0.4)
Divorced	8 (0.8)
Widowed	59 (5.8)
Cohabitation	2 (0.2)
Others	1 (0.1)
Educational period	
0 years	36 (3.6)
<6 years	49 (4.8)
<9 years	365 (36.0)
<12 years	216 (21.3)
12 years	289 (28.5)
>12 years	50 (4.9)
Vocational school graduates (categorized apart from the regular educational course)	8 (0.8)
Farming period	
<30 years	476 (47.0)
≥30 years	537 (53.0)
Types of farming	
Dry fields farming	420 (41.5)
Greenhouses farming	311 (30.7)
Rice farming	157 (15.5)
Orchards farming	125 (12.3)
Annual income, Won	
<20 million	368 (36.3)
20–30 million	209 (20.6)
30–40 million	143 (14.1)
40–50 million	88 (8.7)
>50 million	118 (11.6)
No response	87 (8.6)
Smoking	
Smoked more than five packs of cigarettes during last year	362 (35.7)
Current smoker	223 (22.0)
Secondhand smoking	
Within family (current)	232 (22.9)
Within family (past)	168 (16.6)
Within workplace (current)	66 (6.5)
Alcohol drinking	
Past alcohol drinking	643 (63.5)
Current alcohol drinking	550 (54.3)

BMI, body mass index; SD, standard deviation.

Values are reported as *n* (%), unless otherwise noted.

Table 4 shows the results of health status assessment. Musculoskeletal pain in multiple areas was reported by 925 subjects (91.3%), and the 1-year prevalence rates were as follows: low back pain (63.8%), leg/foot pain (43.3%), shoulder pain (42.9%), wrist/hands/finger pain (26.6%), arm/elbow pain (25.3%), and neck pain (21.8%). Farmers’ stress was above the median value of the Farmer’s Stress Inventory (mean [SD], 77.7 [10.2]; range, 28–112) and Subjective Stress Index (mean [SD], 5.3 [2.4]; range, 0–10), reflecting a stressful status, while the EuroQol-5D-3L index and the EuroQol-Visual Analog Scale scores were high (mean [SD], 0.9 [0.1]; range, −0.171–1 and mean [SD], 67.7 [18.7]; range, 0–100, respectively), reflecting good life quality and subjective health status. Regarding frailty, 174 (17.2%) subjects were classified as frail or pre-frail.

**Table 4.** Health characteristics of enrolled subjects (*n* = 1013)

Variable	
Mean (SD) Farmer’s Stress Inventory (scored 28–112)	77.7 (10.2)
Labor intensity (scored 5–20)	13.7 (3.0)
Job environment (scored 4–16)	11.3 (2.2)
Job esteem and social reputation (scored 2–8)	5.6 (1.4)
Physical environment and weather condition (scored 3–12)	8.8 (1.8)
Financial problems (scored 6–24)	16.0 (2.9)
Uncertainty (scored 2–8)	5.0 (1.3)
Government policy (scored 4–16)	12.2 (1.7)
Health problems (scored 2–8)	5.3 (1.4)
Mean (SD) Subjective Stress Index (scored 0–10)	5.3 (2.4)
Frailty	
Mean (SD) Korean Frailty Index (scored 0–8)	1.3 (1.3)
Level of frailty	
Frailty (scores 5–8)	28 (2.8)
Pre-frailty (scores 3–4)	146 (14.4)
WNL (scores 0–2)	839 (82.8)
Musculoskeletal pain incidence, multiple check	
More than one area	925 (91.3)
Neck	221 (21.8)
Shoulder	435 (42.9)
Arm/elbow	256 (25.3)
Hand/wrist/finger	269 (26.6)
Low back	646 (63.8)
Leg/foot/toe	439 (43.3)
Mean (SD) EuroQol-5D-3L index (range, −0.171–1)	0.9 (0.1)
Mean (SD) EuroQol-Visual Analog Scale (range, 0–100)	67.7 (18.7)

SD, standard deviation; WNL, within normal limits.

Values are reported as *n* (%), unless otherwise noted.

Medical diagnoses are shown in Table 5. Cardiovascular disease was the most frequently reported disease (28.8%), followed by musculoskeletal disease (26.5%) and gastrointestinal disease (25.6%). Of those reporting a history of cardiovascular disease, patients with hypertension comprised the highest proportion (26.0%). For musculoskeletal disease, arthritis comprised the highest proportion (16.5%).

**Table 5.** Medical history of diagnosed diseases (*n* = 1013^a^)

Diagnosed disease	*n* (%)
Cardiovascular disease	292 (28.8)
Hypertension	263 (26.0)
Myocardial infarction	17 (1.7)
Angina	11 (1.1)
Congestive heart failure	2 (0.2)
Arrhythmia	7 (0.7)
Central neurologic disease	24 (2.4)
Stroke	10 (1.0)
Parkinsonism	1 (0.1)
Convulsion	1 (0.1)
Head trauma	3 (0.3)
Endocrinal disease	235 (23.2)
Hyperlipidemia	126 (12.4)
Hyperthyroidism	14 (1.4)
Hypothyroidism	11 (1.1)
Diabetes mellitus	101 (10.0)
Neoplastic disease	31 (3.1)
Respiratory disease	53 (5.2)
Asthma	18 (1.8)
Chronic bronchitis	5 (0.5)
Chronic obstructive pulmonary disease	4 (0.4)
Pneumonia	3 (0.3)
Pulmonary tuberculosis	18 (1.8)
Gastrointestinal disease	259 (25.6)
Gastritis	156 (15.4)
Gastroesophageal reflux disease	54 (5.3)
Hemorrhoid	33 (3.3)
Liver cirrhosis	1 (0.1)
Biliary calculus	15 (1.5)
Hepatitis	18 (1.8)
Genitourinary disease	58 (5.7)
Renal insufficiency	4 (0.4)
Benign prostate hyperplasia	25 (2.5)
Urinary tract infection	4 (0.4)
Musculoskeletal disease	268 (26.5)
Arthritis	167 (16.5)
Osteoporosis	76 (7.5)
Fall	26 (2.6)
Fracture	54 (5.3)

^a^Individuals could have multiple diagnosed diseases.

## DISCUSSION

The FARM study is a population-based cohort study that was developed to assess the musculoskeletal health status and related sociodemographic and agricultural work-related risk factors in Korean farmers. A previous systemic review^4^ showed that lifetime prevalence of any form of MSD among farmers was 90.6%, while 1-year MSD prevalence was 76.9%. Additionally, lifetime low back pain prevalence was 75%, while 1-year low back pain prevalence was 47.8%. We found self-reported prevalence of MSDs in multiple body parts (91.3%), and the most frequent pain site was the low back.

Regarding the characteristics of the study participants compared to overall estimates for farmers in Kangwon Province in 2010,^11^ the sex ratio was similar (47.3% men and 52.7% women in our study vs 49.7% men and 50.3% women in Kangwon province), while the proportions aged ≥65 years (21.2% vs 33.4%) and married (91.1% vs 72.5%) were different because our study recruited farmers aged 40–70 years in order to improve representativeness of active agricultural workers.

Agricultural work involves labor-intensive practices and is related to a multitude of MSD risk factors.^1^ There have been some interventions to reduce the demand for labor-intensive practices^21^ and to improve working efficacy in an effort to reduce the risk of MSDs.^21^^,^^22^ Nevertheless, most Korean farmers suffer due to the harsh working conditions and agricultural work-related MSDs, and there have been few scientific studies to show the actual health status of farmers and the association of MSDs with agricultural workload. Kangwon Province of South Korea is a mountainous area with a high proportion of agricultural workers and a majority of farms that are small in size and that lack access to agricultural machinery. The FARM study may provide information regarding typical farming conditions with high physical demand in Korea.

The purpose of this study is not only to investigate farmers’ baseline characteristics related to MSDs but also to encourage efforts among health professionals, epidemiologists, engineers, and government organizations to reduce incidence of MSDs among farmers. Based on the data acquired, we may report the farmers’ health statuses and trends, and the associations of farmers’ health statuses with sociodemographic and agricultural work-related risk factors. This data will further identify exposures and contribute to the development of valid interventions for agricultural populations using a multidisciplinary approach. The FARM study may also provide a scientific basis for developing interventions and preventive strategies in Korean farmers.
